# Enhancing self-regulation as a strategy for obesity prevention in Head Start preschoolers: the growing healthy study

**DOI:** 10.1186/1471-2458-12-1040

**Published:** 2012-11-30

**Authors:** Alison L Miller, Mildred A Horodynski, Holly E Brophy Herb, Karen E Peterson, Dawn Contreras, Niko Kaciroti, Julie Staples-Watson, Julie C Lumeng

**Affiliations:** 1 Center for Human Growth and Development, University of Michigan, Ann Arbor, USA; 2 School of Public Health, University of Michigan, Ann Arbor, USA; 3 College of Nursing, Michigan State University, Ann Arbor, USA; 4 Department of Human Development and Family Studies, Michigan State University, Ann Arbor, USA; 5 Health and Nutrition Institute, Michigan State University, Ann Arbor, USA; 6 Department of Pediatrics, University of Michigan, Ann Arbor, USA

**Keywords:** Obesity prevention, Low-income children, Preschoolers, Head start, Incredible years series, Self-regulation, Intervention study

## Abstract

**Background:**

Nearly one in five 4-year-old children in the United States are obese, with low-income children almost twice as likely to be obese as their middle/upper-income peers. Few obesity prevention programs for low-income preschoolers and their parents have been rigorously tested, and effects are modest. We are testing a novel obesity prevention program for low-income preschoolers built on the premise that children who are better able to self-regulate in the face of psychosocial stressors may be less likely to eat impulsively in response to stress. Enhancing behavioral self-regulation skills in low-income children may be a unique and important intervention approach to prevent childhood obesity.

**Methods/design:**

The Growing Healthy study is a randomized controlled trial evaluating two obesity prevention interventions in 600 low-income preschoolers attending Head Start, a federally-funded preschool program for low-income children. Interventions are delivered by community-based, nutrition-education staff partnering with Head Start. The first intervention (*n* = 200), Preschool Obesity Prevention Series (POPS), addresses evidence-based obesity prevention behaviors for preschool-aged children and their parents. The second intervention (*n* = 200) comprises POPS in combination with the Incredible Years Series (IYS), an evidence-based approach to improving self-regulation among preschool-aged children. The comparison condition (*n* = 200) is Usual Head Start Exposure. We hypothesize that POPS will yield positive effects compared to Usual Head Start, and that the combined intervention (POPS + IYS) addressing behaviors well-known to be associated with obesity risk, as well as self-regulatory capacity, will be most effective in preventing excessive increases in child adiposity indices (body mass index, skinfold thickness). We will evaluate additional child outcomes using parent and teacher reports and direct assessments of food-related self-regulation. We will also gather process data on intervention implementation, including fidelity, attendance, engagement, and satisfaction.

**Discussion:**

The Growing Healthy study will shed light on associations between self-regulation skills and obesity risk in low-income preschoolers. If the project is effective in preventing obesity, results can also provide critical insights into how best to deliver obesity prevention programming to parents and children in a community-based setting like Head Start in order to promote better health among at-risk children.

**Trial registration number:**

Clinicaltrials.gov Identifier: NCT01398358

## Background

The Institute of Medicine [1] identifies childhood obesity as an urgent problem; nearly 1 in 5 young children are obese and socioeconomic and racial/ethnic disparities in obesity rates appear even during the preschool years [2-4]. Once established, childhood obesity typically persists [5] and predicts health problems including cardiovascular disease and diabetes [6-8]. Preventing obesity is essential, yet few programs for young children have been rigorously tested, and effects are modest [9-11]. The goal of the Growing Healthy trial is to test the effectiveness and feasibility of two obesity prevention interventions. The first intervention is an education curriculum promoting obesity prevention behaviors, and the second intervention places this curriculum in the context of an additional intervention to enhance behavioral self-regulation. Both interventions are delivered to low-income preschoolers and their parents by community-based, nutrition-education specialists partnering with Head Start (HS) staff.

The education curriculum, Preschool Obesity Prevention Series (POPS), is designed to promote empirically-tested obesity prevention behaviors informed by expert recommendations by the American Academy of Pediatrics (AAP) [12] and the American Dietetic Association (ADA; now Academy of Nutrition and Dietetics) guidelines for Pediatric Weight Management [13]. The ADA supports increased fruit and vegetable (FV) intake based on intervention trials that found FV intake associated with reduced adiposity indices [14-16]. Sugar-sweetened beverage (SSB) consumption is consistently associated with body mass index (BMI) and child obesity in prospective studies and intervention trials, [12,13,17-21] and inversely associated with milk intake [22]. Dietary variety is related to greater FV intake and dietary quality [23]. The Centers for Disease Control (CDC) [24] and AAP recommend that screen time be limited to less than 2 hours per day, [12] based on intervention trials [25-27] and observational studies [25]. Thus, POPS focuses on increasing FV intake, reducing SSB consumption and eating a diet rich in calcium (following United States Department of Agriculture (USDA) recommendations on low-fat dairy), increasing dietary variety, and reducing screen time. POPS involves both children and parents in learning about obesity prevention behaviors and building skills to enact them, which is critical for a comprehensive approach, yet not common in existing programs.

Improving behavioral self-regulation (i.e., inhibiting impulses, calming down when upset) may also be important for preventing childhood obesity. Stress can lead to increased appetite and a shift in preference to foods high in added sugar and fats [28-30]. Obesity is more common in children who engage in emotional eating, [31,32] disinhibited eating, [33] or eating in the absence of hunger [34-36]. Children who cannot cope with stress well may engage in such stress-eating patterns as a way to self-regulate emotion and behavior, and over time become obese. The literature supports this impression; children who have difficult temperaments, [37-39] behavior problems, [40] tantrums over food, [41] impulsivity, [14,42-45] and difficulty delaying gratification [46-51] are more likely than their peers to be obese. The preschool years are an important period for the development of eating behavior, [52] lifelong obesity risk, [53] and self-regulation [54]. Thus, developing effective interventions for children this age that integrate these domains – and, importantly, include parents – could be a promising new direction for obesity prevention.

**Figure 1:**
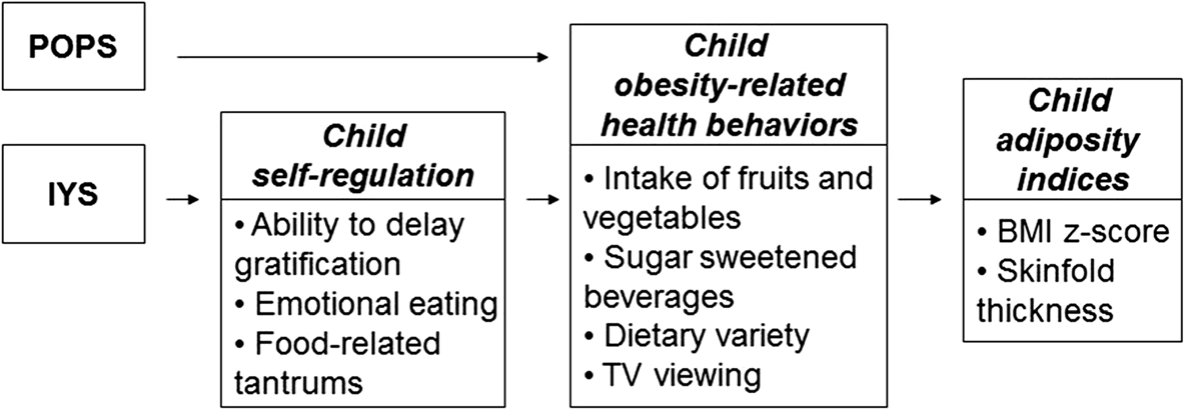
Conceptual model.

The Growing Healthy Study tests this novel combined approach to obesity prevention. First, the POPS intervention is designed to provide developmentally appropriate, empirically-validated, and coordinated obesity prevention messages to preschoolers and their parents. Second, we embed POPS within a self-regulation framework by implementing the Incredible Years Series (IYS), an evidence-based program shown to enhance self-regulation in young, low-income children [55-57]. With an emphasis on modeling positive behavioral management techniques and parenting as part of IYS, the IYS + POPS intervention will teach children behavioral self-regulation strategies (e.g., ways to calm down without relying on food), and also give parents new techniques for managing child behavior (e.g., responding to children’s tantrums in ways that do not involve food). Thus, the IYS + POPS intervention may not only prevent obesity through directly reducing stress-eating as a form of self-regulation, but also lay the groundwork for better overall ‘absorption’ of information in the POPS curriculum by enhancing child self-regulation and improved parent–child interactions. Our conceptual model is outlined in Figure 1.

### Study aims and hypotheses

The primary aim of this study is to examine the effectiveness and feasibility of the two obesity prevention interventions described above: 1) POPS and 2) POPS + IYS, as delivered by community-based nutrition educators and HS staff to low-income preschoolers and their parents. Hypotheses are that across an academic year, compared to Usual HS Exposure: (1) POPS will result in improved obesity-related health behaviors and adiposity indices; (2) POPS + IYS will result in the greatest improvement in obesity-related health behaviors and adiposity indices, and this greater effect will be mediated in part by increased child self-regulatory capacity. A process evaluation to assess the feasibility, fidelity, parental engagement, and educational effectiveness of the POPS and POPS + IYS interventions will also be conducted. This project was funded by the USDA National Institute of Food and Agriculture (NIFA) Agriculture and Food Research Initiative (AFRI) Competitive Grants Program, Childhood Obesity Prevention Challenge Area.

## Methods/design

### Study design

The Growing Healthy study is a randomized controlled trial (RCT). Classrooms (*n* = 75) are randomized through an automated system to one of 3 study arms (200 children per arm): (1) POPS; (2) POPS + IYS; or (3) Usual HS Exposure. Each HS program hosts all 3 study arms, and all children in an individual classroom participate in only one arm. Only one classroom participates per physical school site to prevent cross-contamination across study arms and ensure that participants are blind to group assignments. Families are assigned to study arm as a function of their classroom assignment. Every effort is made to match intervention and comparison classrooms on demographic characteristics in order to minimize differences not due to the intervention (e.g., % minority race/ethnicity, teacher education).

Data are collected pre-intervention (in the fall) and post-intervention (in the spring). Interventions occur between October and April of the academic year. Process implementation data are collected throughout the study. Children are enrolled in each of 4 academic years, with 50 children enrolled into each of the 3 study arms per year. Data collectors are separate from the interventionists, and blinded to the study arm to which participants in a classroom are assigned.

### Participants and recruitment

Participants are from three HS programs in mixed rural and urban areas of Michigan. These programs serve 3,222 children (67% white, 28% black; ethnicity 23% Hispanic; 12.6% English as second language) per year in 184 half-day classrooms. The prevalence of overweight (BMI ≥ 85th percentile and < 95th, based on US Centers for Disease Control (CDC) reference growth curves for age and sex), 16.2%) and obesity (BMI ≥ 95th percentile, 17.0%) in these programs is similar to other HS cohorts [3,58]. Inclusion criteria are that the child is aged 3 or 4 years at study enrollment. Exclusions are significant developmental disabilities that would preclude participation, child is a foster child, or parent is non-English-speaking. Almost all families in the participating HS programs have annual household incomes below the federal poverty line, as this is an eligibility requirement for 90% of the children enrolled.

Families are told about the study during classroom open houses and through flyers in children’s backpacks, and compensated for returning an initial enrollment packet, including a signed written informed consent form. They are then contacted by telephone to review eligibility criteria that they reported in the enrollment packet and to confirm complete understanding of the study and validate informed consent. Next, a data collector (blind to study condition) completes pretest assessments. Appropriate compensation, tokens of appreciation and regular contact from project staff are incorporated to enhance retention.

### Research ethics approval

This study has been approved by the Institutional Review Boards of both collaborating universities, the University of Michigan Medical School Institutional Review Board (UM-IRBMED) and Michigan State University Social Science / Behavioral Education Review Board (MSU-SIRB). We follow the usual standards of a Data and Safety Monitoring Plan as required by the National Institutes of Health for an RCT. Quality assurance reports are prepared on a monthly basis and reviewed by the Study Directors.

### Interventions: POPS and POPS + IYS

#### Preschool obesity prevention series (POPS)

Social Cognitive Theory was the theoretical basis for the curricula that have informed POPS (Healthy Toddlers [59]; Nutrition Education Aimed at Toddlers [60]). POPS also uses an Experiential Learning approach, which emphasizes active learning through the senses. Each lesson provides opportunities to engage with content (e.g., cooking activity), reflect on content through discussion, and apply the new concept (e.g., through goal setting). POPS parent and child sessions include complementary content to reinforce concepts and improve parental knowledge and efficacy to promote specific childhood obesity prevention behaviors. Content of units is summarized in Table 1 (each unit contains more than one lesson).

**Table 1:** POPS Units

**POPS-Parent**	
**1**	Eating a Rainbow of Fruits and Vegetables
**2**	Trying New Foods: From Never to Maybe
**3**	Turning Off the TV and Tuning Into Fun and Family
**4**	Keeping You and Your Child Naturally Sweet – Limiting Sugar-Sweetened Beverages through Role-Modeling
**5**	Let’s Make Easy and Healthy Meals at Home
**6**	Eating Together: Family Meals = Better Diets
**7**	A Healthy Way To Start The Day: Meal Planning
**8**	Healthy Choices When Eating Out
**POPS-Child**	
**1**	Eating the Alphabet
**2**	I Will Never Not Ever Eat a Tomato
**3**	The Berenstain Bears and Too Much TV
**4**	Oliver’s Milkshake
**5**	It’s a Sandwich
**6**	My Amazing Body

The POPS-Parent component consists of eight 75-minute weekly lessons followed by reinforcing telephone contacts after every other lesson. Each lesson offers opportunities for parents to develop and practice skills, and a discussion of strategies to overcome challenges and problem-solving techniques, with an emphasis on building knowledge and self-efficacy about preventing childhood obesity. Recipes and hands-on activities are included in each lesson. Lessons are implemented by community-based, master’s level trained Extension Educators from Michigan State University (a land-grant institution). Consistent with the land-grant mission to translate research findings into community-based practices, Extension Educators help people improve their lives through an educational process that applies knowledge to critical issues, needs and opportunities, Extension Educators regularly collaborate with scholars and work with parents, schools and community organizations, and each POPS Educator has extensive experience working with parents in the community on nutrition education issues.

The POPS-Child component uses children's stories with embedded obesity prevention themes related to behavioral goals (e.g., more FV consumption; less screen time). Six lessons are delivered over 12 weeks by the HS teacher and Extension Educators. Activities include classroom cooking experiences, games/activities associated with story themes, and goal setting. "Family Links" and "Parent Pages" are sent home to reinforce content from school to home. Each lesson runs on a two-week cycle in which the classroom teachers read the selected book (featuring one or more of the POPS obesity prevention behaviors) and introduce reinforcing classroom activities. The following week, the Extension Educator revisits the story and implements a hands-on cooking/tasting activity with the children.

#### POPS training, quality control, and implementation fidelity

To ensure consistency in the implementation of the curriculum, we conduct a 2-day training for Extension Educators and a shorter training for HS teachers (with booster sessions each year). Training covers curriculum specifics as well as strategies for promoting parent self-efficacy for behavior change and importance of fidelity. Fidelity of POPS delivery in the field is assessed by observing at least one session per group (including classroom activities and parent sessions) and documenting any deviations from the curriculum. Phone support from trainers is also available as needed. Child and parent engagement in POPS classroom and parent sessions, respectively, are documented by extension educators.

### Incredible Years Series (IYS)

IYS [56] is a widely-used, evidence-based program that includes Parent, Teacher, and Child components designed to promote self-regulation and prevent behavior problems in children. IYS uses observational learning and reinforcement techniques, and emphasizes behavior change strategies such as descriptive commenting about child behavior, praise, role-plays, and coaching to encourage and model positive behavior for parents and children [61]. Effects are greatest when multiple components (Parent, Child, Classroom) are implemented, [56] which is our approach. IYS has been extensively tested and found to be effective with HS children and families [55,57]. IYS units are listed in Table 2 (each unit includes multiple lessons).

**Table 2:** IYS Units

**IYS-Parent (BASIC)**	
**1**	Strengthening Children’s Social Skills, Emotional Regulation & School Readiness
**2**	Using Praise and Incentives to Encourage Cooperative Behavior
**3**	Positive Discipline – Rules, Routines and Effective Limit Setting
**4**	Positive Discipline – Handling Misbehavior
**IYS-Child (Dinosaur School)**	
**1**	Making Friends and Learning Rules (Apatosaurus)
**2**	Understanding and Detecting Feelings (Triceratops)
**3**	How to Do Your Best in School (Iguanodon)
**4**	Problem-Solving Steps (Stegosaurus)
**5**	Anger Management (Tyrannosaurus Rex)
**6**	How to Be Friendly (Allosaurus)
**7**	How to Talk With Friends (Brachiosaurus)
**IYS-Teacher (Classroom Management)**	
**1**	The Importance of Teacher Attention, Encouragement, Praise
**2**	Motivating Children Through Incentives
**3**	Preventing Behavior Problems—the Proactive Teacher
**4**	Decreasing Students’ Inappropriate Behaviors
**5**	Building Positive Relationships With Students, Problem Solving

The IYS-Parent component (12–14 weeks, 2 hours/week) focuses on parenting skills such as using effective praise, incentives, limit-setting, and handling misbehavior. Concepts are discussed using video vignettes about parenting challenges. Parents complete homework and receive follow-up phone calls. IYS-Parent sessions are delivered by master’s-level mental health specialists (MHS) (one per HS program, employed by HS).

For the IYS-Child component, sixty 15–20 minute lessons are delivered throughout the year during "Circle Time" in HS classrooms, followed by small group activities. Lessons address self-regulation skills, problem-solving strategies, and prosocial behavior, and use child-size puppets to teach skills and engage children. IYS-Child lessons are delivered by the MHS, and HS teachers direct small group activities after each lesson. Teachers are mentored by MHS in delivering IYS-Child, so that delivery can slowly progress from delivery by MHS, to co-delivery by MHS and teacher, to sole delivery by the teacher over time, which is an effective model for dissemination [62]. MHS and teachers also receive training in classroom management strategies (e.g., handling transitions effectively), which is the IYS-Classroom component.

### IYS training, quality control and implementation fidelity

MHS and teachers receive extensive training in all components that they deliver, as required by IYS [56]. MHS communicate extensively to share ideas and coordinate efforts across sites, and receive monthly supervision from IYS trainers about their delivery of IYS components, and consultation as needed. MHS also work with teachers within their own site to develop lesson plans and small group activities. Fidelity of IYS intervention delivery is assessed via videotape, and covers the same elements as our POPS fidelity assessment.

### Comparison group: “Usual Head Start Exposure”

In the Usual HS Exposure group, classes are taught by a teacher and teacher’s aide. HS performance standards require nutrition and parenting curricula, but not intensive programs like POPS or IYS. Nutrition information is delivered to parents primarily via newsletter and each HS program contracts with a dietitian to review menus and individual child needs; these activities are documented for all programs.

### Data collection procedures

Data collectors (blind to family intervention status and not involved in implementation) are trained by Study Directors in all protocols. Data collection quality is monitored (e.g., surveys completed fully; child weight/height in appropriate range). Data collectors are trained to respond appropriately with referral information if parents make such requests.

Prior to intervention start in the fall, data collectors make a home visit to measure maternal weight and height, gather questionnaires, and obtain the first of three 24-hour dietary recalls (24HR; remaining 24HR are completed by phone). Children are weighed, measured, and participate in a 15-minute videotaped self-regulation task in a private room at school. Teachers complete questionnaires for each child. In the spring, mothers, children, and teachers repeat the same tasks as post-intervention assessments.

Questionnaires are completed using computer-assisted interviewer administration. Data are saved to a master database after each home visit, or double entered and any data entry errors corrected. Video-recordings of self-regulation tasks (see below) are coded by undergraduates trained to reliability (Cohen’s kappa ≥ 0.70) by Study Directors. Data are stored on a password protected server, which is automatically backed up multiple times per day.

### Primary outcome measures

#### Anthropometry

Individuals are weighed without shoes or heavy clothing using a Detecto Portable Scale Model #DR550C and measured using a Seca 213/217 portable stadiometer. Duplicate measures are taken for both weight and height. Body mass index (BMI) is calculated and child BMI z-score derived (using CDC reference growth curves for age and sex [63]), in order to estimate prevalence of child obesity and overweight (classified per national recommendations [12]). Maternal weight and height are measured using the same methods, and maternal BMI calculated (kg/m^2^) for use as a covariate.

Triceps and subscapular skinfolds improve prediction of body fatness and correlate with chronic disease risk in children [64,65]. Duplicate measures of each skinfold in children are obtained using Lange Calipers (triplicates when tolerance of 2 mm is exceeded) [15,64].

#### Obesity-related health behaviors

Three 24-hour dietary recalls (24HR) are collected from mothers regarding child intake, using the USDA 5-step Automated Multiple Pass Method [66]. The initial 24HR is obtained at the home visit, using food models and handouts showing child-appropriate portion sizes to assist [67-69]. Two additional unannounced 24HR are conducted by phone. The 24HRs generate data on individual foods [70] and food groups, [71] including daily FV and SSB servings, adjusting for energy (kcal) intake [17,72]. We also assess dietary variety based on these data.

Screen Time is based on maternal report of the child’s TV viewing and computer use on weekdays and weekends [27,67,73]. Maternal report of usual minutes of outdoor play, a covariate, is assessed with a validated measure for preschoolers [74].

#### Food-related self-regulation

Ability to Delay Gratification (ATDG) is a well-validated measure of self-regulation [75] that tests a child’s ability to wait when an appealing food is presented. Videotapes of this task are later coded to assess how long the child waits to eat the treat, and what behaviors they use during the waiting period (e.g., how much they pay attention to the treat vs. ignore it).

Emotional Eating is measured using the Children’s Eating Behavior Questionnaire, [76] a validated and reliable maternal-report questionnaire with 8 subscales: Food Responsiveness, Emotional Overeating, Enjoyment of Food, Desire to Drink, Satiety Responsiveness, Slowness in Eating, Emotional Undereating, and Food Fussiness Undereating, and Food Fussiness.

Food-related tantrums are captured with a series of questions used in prior work [41]. Mothers report, during the last 4 weeks, how often (1) the child asked for something to eat; (2) the mother told the child he/she could not have something to eat; (3) the child became upset in response; (4) the child had a tantrum in response.

#### Parent knowledge and self-efficacy

Change in parent knowledge, self-efficacy, and outcome expectations regarding child nutrition and obesity prevention is assessed using questionnaires specific to POPS, and knowledge and self-efficacy regarding parenting and child behavior is assessed with questionnaires developed by IYS.

### Secondary outcome measures, covariates, and demographics

Additional constructs that may relate to obesity and/or self-regulation (e.g., maternal depression; child temperament) are also assessed. Parents complete the Caregivers’ Feeding Styles Questionnaire, [77] Dutch Eating Behavior Questionnaire, [78] USDA Household Food Security Scale, [79] Center for Epidemiologic Studies Depression Scale, [80] Child Behavior Questionnaire-Short Form, [81] The Parenting Scale, [82] and the Alabama Parenting Questionnaire [83]. Parents and teachers both complete the Eyberg Child Behavior Inventory (Student Behavior Inventory version for teachers) [84]and the Social Competence and Behavior Evaluation [85]. Following ATDG, children participate in Gift Delay- Wait, Bow and Wrap Tasks [86] to assess non-food-related self-regulation, and in brief interviews to assess their emotion knowledge [87], a focus of IYS. Because an element of each intervention is classroom-based and results may reflect classroom-level processes, independent observers also assess the emotional climate of the classroom and teachers’ support of children’s self-regulation (fall and spring) via the Caregiver Interaction Scale [88] and the Supports for Social-Emotional Growth Assessment [89]. Teachers also report their use of IYS classroom management strategies using the Teacher Strategies Questionnaire [55]. Finally, parents provide demographic data, including child sex, race, ethnicity, maternal education, family income-to-needs ratio, family structure, and child’s birth weight and gestational age.

### Process evaluation measures

Process data is gathered to assess intervention implementation and feasibility. Data include parent attendance at sessions, parent satisfaction with program elements, and reasons for not attending sessions or withdrawing from the study (via exit interviews). Child school attendance and participation in the lessons is also recorded.

In addition to implementation fidelity observations (described above), HS Staff and Extension Educators complete questionnaires regarding intervention buy-in (e.g., perceived need for and interest in the intervention, time devoted to implementation), and are interviewed on a quarterly basis regarding intervention strengths/weaknesses, implementation issues, and challenges of retention.

### Sample size, power calculations, and data analysis

#### Sample size and power calculations

Enrolling 200 participants per arm, assuming attrition of 25%, will achieve a final sample size of 150 participants per arm. This sample size of 150 per arm will enable detection of small to moderate effect sizes of f = .16 (between groups vs. within group variation), with a power of 80%, α = .05 and assuming and intraclass correlation *r* = .05. This sample size also provides 80% power with *r* = .20, α = .05 to detect an effect size of f = .185.

#### Analysis plan

The primary objective is to determine whether POPS + IYS is more effective, compared to POPS alone or Usual HS Exposure, in reducing adiposity measures (BMI z-score, skinfolds thickness). Additional focal outcomes are dietary intake, TV viewing, ATDG, emotional eating, and food-related tantrums. Baseline comparability of the 3 groups will be assessed. To account for clustering of children within a classroom, Proc Mixed in SAS with a random intercept for classroom will be used to analyze continuous outcomes, and General Estimating Equations (GEE) techniques [90] will be used to compare the prevalence of obesity and overweight across the 3 groups. Analyses will be conducted both with and without adjustment for baseline characteristics such as age, sex, race, and baseline BMI or BMI z-score. All models will incorporate covariates that are related to the outcomes to reduce residual variance and thus help reduce Type II error. Significance will be assessed using a two-sided test at α = 0.05.

Analyses will be based on the intention to treat principle where all randomized participants, including dropouts, are included in the analysis based on their randomized intervention group [90]. Given that a substantial portion of participants will likely have only partial adherence to the intervention (e.g., only attend some parent sessions), dose–response analyses will also be performed where dose corresponds to number of sessions attended.

As outlined in the conceptual model (Figure 1), analyses will be conducted to test whether the effect of POPS + IYS on obesity-related health behaviors and adiposity indices is mediated by self-regulation (using parent-reported, teacher-reported, and direct child assessment measures). As a secondary analysis, models will also be examined separately for boys and girls, white and non-white children, and children who are overweight or obese versus non-overweight at baseline.

Finally, process data on recruitment, retention, participant engagement in the intervention, participant and educator satisfaction, and implementation fidelity will be analyzed using descriptive and qualitative approaches, to inform continued collaborations with HS and allow more effective dissemination of findings.

## Discussion

The Growing Healthy study will provide: 1) increased knowledge about self-regulation promotion as a strategy for obesity prevention in preschoolers, which will add to the scientific literature; 2) demonstration of the feasibility of an intervention delivered by HS staff and Extension educators; and 3) a research-based, empirically-tested obesity prevention curriculum product appropriate for use by HS and Extension staff. Thus, the study should advance fundamental science as well as translational research by generating new knowledge of the behavioral factors that influence childhood obesity, and by delivering this science to people who serve children through education and extension efforts.

POPS and POPS + IYS are theory-based, multi-component interventions that have the potential to be sustainable, given that they are being implemented in existing infrastructures and by community-based educators. By providing POPS and IYS in partnership with such agencies, the potential exists to enhance programming nationwide through broad-based dissemination. Analysis of the effective components of the POPS and POPS + IYS interventions may thus have important implications not only for early childhood curricular practices within Head Start but also in other early childhood programs.

## Abbreviations

POPS: Preventing Obesity in Preschoolers Series; IYS: Incredible Years Series; USDA: United States Department of Agriculture; NIFA/AFRI: National Institute of Food and Agriculture/Agriculture and Food Research Initiative; HS: Head Start; AAP: American Academy of Pediatrics; ADA: American Dietetic Association; FV: Fruit and vegetable; SSB: Sugar-sweetened beverage; BMI: Body mass index; CDC: Centers for Disease Control; RCT: Randomized controlled trial; UM: University of Michigan; MSU: Michigan State University; MHS: Mental health specialist; 24HR: 24-Hour dietary recall; ATDG: Ability to Delay Gratification.

## Competing interests

The authors declare that they have no competing interests.

## Authors’ contributions

ALM, JCL, MAH, HBH, KEP, and DC conceived the project, contributed to the development of the study design and obtained funding, developed the interventions and conceived of the study design, and drafted the manuscript. NK carried out the statistical analyses and design and revised the manuscript. JSW managed the project and monitored recruitment and intervention procedures. All authors read and approved the final manuscript.

## Pre-publication history

The pre-publication history for this paper can be accessed here:

http://www.biomedcentral.com/1471-2458/12/1040/prepub
